# Sex‐Specific Differences in Symptomatology and Quality of Life Impact of Allergic Rhinitis: A Cross‐Sectional Study

**DOI:** 10.1002/iid3.70565

**Published:** 2026-10-22

**Authors:** Hasan Ibrahim Al‐Balas, Almu'atasim Khamees, Shatha Khaled Al Saoud, Sajeda Ahmad Aldakhiel, Wasan Abdallah Bani Issma'eel, Farah Nayef Alzu'bi, Nour Ahmad Mistareehi, Majd Mohammed Qashou', Sireen Kamal Ayasrah

**Affiliations:** ^1^ Faculty of Medicine Yarmouk University Irbid Jordan; ^2^ Ministry of Health Irbid Jordan

**Keywords:** adult, allergic rhinitis, psychosocial burden, quality of life, sex differences

## Abstract

**Background:**

Allergic Rhinitis (AR) is a widespread condition that significantly affects the quality of life (QOL) of those affected globally. It contributes to sleep disturbances, fatigue, poor concentration, irritability, and can worsen symptoms in individuals with coexisting asthma. This study examines the impact of AR on QOL in adults, with specific attention to sex differences.

**Materials and Methods:**

A cross‐sectional online survey using a purpose‐designed, non‐validated questionnaire was distributed via social media using convenience sampling to 1572 Jordanian adults. The questionnaire collected data on demographics, AR symptoms, triggers, and the impact of AR on mental health, social functioning, and daily life. Data were analyzed using descriptive statistics and Chi‐square tests. Multivariable logistic regression was used to adjust for potential confounders, and Benjamini–Hochberg false discovery rate (FDR) correction was applied to account for multiplicity.

**Results:**

A total of 1572 respondents participated; 683 (43.4%) had physician‐diagnosed AR. AR was reported by 157/419 males (37.5%) and 526/1153 females (45.6%); this sex difference was statistically significant (*χ* ^2^ = 8.31, *p* = 0.004; OR = 1.40, 95% CI 1.11–1.76). Most patients lived in urban areas (69.4%). While sneezing and rhinorrhoea were common across sexes, females reported significantly higher rates of nasal itching/congestion (55.5% vs. 43.9%, *p* = 0.011), headaches (61.0% vs. 43.9%, *p* < 0.001), and increased stress/anxiety (53.4% vs. 42.7%, *p* = 0.018, not surviving false discovery rate (FDR) correction). In contrast, snoring was more prevalent in males (40.1% vs. 24.1%, *p* < 0.001). Regarding QOL, AR severely disrupted sleep (80.7%) and concentration (66.2%) for both sexes. However, females were significantly more likely to report irritability/emotional distress (65.4% vs. 55.4%, *p* = 0.023), prefer social isolation during symptoms (59.3% vs. 47.8%, *p* = 0.010), and experience limitations in activities outside the home (55.1% vs. 45.9%, *p* = 0.041). After adjustment, female sex remained significantly associated with irritability/emotional distress (aOR = 1.50, 95% CI 1.02–2.22, *p* = 0.039) and preference for social isolation (aOR = 1.53, 95% CI 1.05–2.23, *p* = 0.028). After Benjamini–Hochberg FDR correction, nasal itching/congestion, snoring, and headache remained statistically significant in symptom analyses; no QOL items survived FDR correction, and these findings should be considered exploratory. Asthma was reported by 80/683 (11.7%) of AR patients, more frequently among males than females (19.7% vs. 9.3%, *p* < 0.001). Sensitivity analyses showed that the association between female sex and social isolation was robust to adjustment for or exclusion of asthma, whereas the association with irritability was attenuated after excluding participants with asthma.

**Conclusion:**

These findings suggest AR's substantial impact on daily functioning and mental well‐being, with signals of greater psychosocial burden among women. Improved understanding of sex‐specific challenges can support more effective prevention, diagnosis, and management strategies, with tailored strategies to address the distinct symptom burden and QOL impairments experienced by female patients. These findings may provide preliminary insights into the burden of AR in Jordan and should be confirmed in representative, validated surveys.

## 1 Introduction

Allergic Rhinitis (AR) is a prevalent atopic disease characterized by bothersome symptoms, including nasal congestion, clear rhinorrhea, sneezing, postnasal drip, and nasal itching. These symptoms can significantly impact individuals, decreasing productivity, increasing healthcare costs, and reducing overall quality of life [1]. In fact, Allergic Rhinitis has become a global health concern, affecting approximately 10%–40% of the population, with wide regional variation and an increasing prevalence over the past 20 years, which causes major illness and disability worldwide [2]. It is a condition that knows no boundaries, affecting individuals from various ethnic backgrounds, across different age groups, and from all corners of the globe [2].

AR develops when the immune system recognizes an environmental allergen and initiates sensitization, during which allergen‐specific IgE binds to the high‐affinity FcεRI receptor on mast cells and basophils. On re‐exposure, cross‐linking of receptor‐bound IgE triggers the release of histamine, leukotrienes, and other mediators, producing the early‐phase response, and recruits eosinophils and other inflammatory cells that sustain the late‐phase response and chronic mucosal inflammation [3]. Historically, AR has been classified as seasonal AR (SAR) and perennial AR based on whether outdoor or indoor allergens predominate. SAR is triggered periodically by outdoor allergens, such as grass, tree, or weed pollen, while perennial AR symptoms persist throughout the year due to indoor allergens like house dust mites, animal dander, insects, and mold [4]. However, this classification did not fully understand the occurrence and manifestations of the disease [5]. Accordingly, the Allergic Rhinitis and its Impact on Asthma (ARIA) workshop proposed a new classification in 2001, dividing AR by symptom duration into Intermittent AR (IAR) and Persistent AR (PAR). The ARIA recommendations were subsequently updated in 2008 to further refine this approach [5]. The severity of allergic rhinitis is categorized as “mild” or “moderate/severe,” taking into account not only symptom presence but also their impact on the individual's quality of life (QOL) [2, 5, 6]. Furthermore, AR is not only a localized nasal disorder but also a systemic inflammatory disease often associated with comorbidities such as asthma, sinusitis, conjunctivitis, atopic disease, and otitis media [2]. Therefore, it is crucial to comprehensively evaluate the quality of life (QOL) of patients affected by AR.

The World Health Organization (WHO) defined quality of life (QOL) as “QOL encompasses psychological and social functioning, as well as physical functioning, and incorporates both positive aspects of well‐being and negative aspects of disease or infirmity.” This definition of QOL has a common basis with the WHO's definition of health, given in 1948. On the other hand, the Quality‐of‐Life Research Unit at the University of Toronto defines QOL as the extent to which a person can enjoy the valued possibilities of their life [7]. In line with this, the present study aims to assess the QOL of Jordanian adults suffering from AR. We evaluated quality of life (QOL) by administering a questionnaire assessing various aspects of QOL, including physical and mental well‐being, social functioning, relationships, educational status, work environment, and overall life satisfaction.

Multiple studies have reported sex differences in the prevalence, clinical presentation, and QOL impact of AR, although findings have been inconsistent and appear to be age‐dependent [1, 5]. A large meta‐analysis of 67 studies found that male children under 11 years were 1.2‐fold more likely to exhibit rhinitis symptoms, but this pattern reversed during adolescence (11–18 years), with females becoming more frequently affected [8]. In adulthood, some studies report a female predominance, while others find no significant difference [5, 9]. For example, a large database analysis from the United States found that female sex was associated with AR among adults but not with non‐allergic rhinitis, suggesting a potential role for sex hormones in the pathogenesis of allergic inflammation [10]. Experimental evidence supports this, showing that female patients with AR have higher sensitivity to irritants and airway hyperresponsiveness than males, and that estrogen can modulate mast cell activation and allergic immune responses [11]. Despite this growing body of literature, data on sex differences in the symptom profile and QOL burden of AR in Middle Eastern populations, including Jordan, remain limited. This gap provided the rationale for the present study, which aims to investigate and compare symptomatology, triggering factors, and the multifaceted impact on QOL between male and female adults with AR in Jordan.

Therefore, this study aims to specifically investigate and compare the symptomatology, triggering factors, and the multifaceted impact on QOL between male and female adults with AR in Jordan. By elucidating these sex‐specific differences, this research seeks to inform more personalized and effective patient care strategies.

## 2 Methods

This cross‐sectional study used an online questionnaire purpose‐designed and developed from a review of established QOL instruments (e.g., the Mini Rhinoconjunctivitis Quality of Life Questionnaire) and clinical expertise. The questionnaire was designed to capture four domains: (1) demographic and clinical characteristics (e.g., age, sex, residence, family history, and smoking status), (2) AR symptomatology and seasonality, (3) common triggering factors (triggers were self‐attributed by participants and were not confirmed by skin‐prick or specific IgE testing; smoking refers to perceived tobacco‐smoke exposure as a trigger and is distinct from current smoking status), and (4) the impact of AR on various QOL aspects (sleep, work, concentration, emotional, and social well‐being). Residence was recorded for all respondents and was therefore available for residence‐specific prevalence calculations. The questionnaire was piloted on a small group (*n* = 20) for clarity and face validity before widespread distribution. It was disseminated in Arabic via social media platforms (e.g., Facebook and WhatsApp) between January and March 2023.

At the beginning of the survey, we stated that the collected data would be used only for this study. The survey questions did not collect personal information, and participants completed it voluntarily and could stop at any time. We excluded all participants below 18 years old, pregnant individuals, and those with psychological problems. Participants with self‐reported psychological problems were excluded because the primary outcomes were psychological and we wished to reduce confounding by pre‐existing psychiatric illness. However, this exclusion may have attenuated the observed psychological associations and limited generalizability.

A brief introduction to the disease was followed by a direct question asking participants whether a physician had diagnosed them with AR. All who were diagnosed by a physician were considered AR patients. The questionnaire also asked whether a physician had ever diagnosed the participant with asthma. Participants self‐reported this information, and we did not independently verify it using medical records, spirometry, or other objective clinical measures. The item was asked of all AR respondents.

Of the 1680 records received by the survey platform, 24 were excluded because they were duplicate submissions, 12 because the respondent was younger than 18 years, nine because the respondent was pregnant, and 14 because of self‐reported psychological problems. A further 125 records were excluded as incomplete. During this revision, we re‐extracted the raw responses directly from the survey platform and identified 76 valid responses inadvertently omitted during the initial extraction; we re‐included them. The final analytic sample comprised 1572 respondents, of whom 683 had physician‐diagnosed AR (157 men and 526 women), and 889 did not. All analyses in this manuscript are based on the re‐audited dataset; the original submission used an earlier extraction of 1526 respondents (663 with AR).

No formal a priori sample‐size calculation was performed because the study used online convenience sampling. The survey was designed so that only participants who reported a physician diagnosis of AR were routed to the sections on AR symptoms, triggers, and the impact of AR on quality of life. Participants who did not report a physician diagnosis of AR were not asked these questions and were directed to the end of the survey. Therefore, non‐AR respondents did not provide data on symptoms, triggers, or QOL impacts.

Data were analyzed using the Statistical Package for the Social Sciences version 25 (SPSS) statistical software. Descriptive statistics are presented as frequencies and percentages. Sex‐stratified categorical comparisons were performed using *χ* ^2^ tests; *χ* ^2^, *df*, and exact *p*‐values are reported for every comparison. Effect sizes are presented as unadjusted odds ratios (OR) with 95% confidence intervals (95% CI). To account for multiplicity, Benjamini–Hochberg false discovery rate (FDR) correction was applied separately to three families of comparisons: symptoms (nine tests), triggers (eight tests), and quality‐of‐life items (15 tests). Findings with FDR‐adjusted *q* < 0.05 were considered statistically significant; others were considered exploratory. The multivariable logistic regression models examining irritability/emotional distress and preference for social isolation were exploratory and were selected following the initial unadjusted analyses because these outcomes showed notable sex differences. The adjusted *p*‐values from these models were not subjected to a separate multiplicity correction and are therefore interpreted cautiously as exploratory adjusted associations rather than confirmatory findings. Multivariable logistic regression was performed for the two headline psychosocial outcomes (irritability/emotional distress and preference for social isolation), adjusting for age, sex, income, occupation, smoking status, family history of AR, urban/rural residence, and education. Adjusted ORs with 95% CIs are reported. No a priori power calculation was performed. The sample was one of convenience. Analyses were exploratory and hypothesis‐generating. A *p*‐value of < 0.05 was considered statistically significant.

Both logistic regression models used complete‐case analysis; no values were missing for the included covariates. All categorical covariates were coded as binary indicator variables with the following reference categories: sex (male), income (≥ 500 JD), occupation (working), smoking (non‐smoker), family history of AR (no), residence (rural), and education (university or higher). The number of events per variable was 31.5 for the irritability model and 37.0 for the social isolation model. Model fit was assessed using the Hosmer–Lemeshow test; collinearity among the covariates was assessed using variance inflation factors, all of which were below 1.48.

## 3 Results

The questionnaire was administered to 1572 individuals, comprising 419 males and 1153 females, with a median age of 35 years (range: 18–76). Of these, 683 (43.4%) participants reported a physician diagnosis of AR. Sex‐specific prevalence was 157/419 men (37.5%) and 526/1153 women (45.6%), a statistically significant difference (*χ* ^2^ = 8.31, *df* = 1, *p* = 0.004; OR = 1.40, 95% CI 1.11–1.76). Out of the AR participants, 21.2% were smokers (145 individuals), while the remaining 78.8% were non‐smokers (538 individuals). Moreover, 382 individuals (55.9%) had a family history, while 301 individuals (44.1%) did not. Among all 1572 respondents, AR prevalence was higher in urban than in rural residents (474/1006, 47.1% vs. 209/566, 36.9%; *χ* ^2^ = 15.31, *df* = 1, *p* < 0.001; OR = 1.52, 95% CI 1.23–1.88). The AR subgroup was predominantly urban (474/683, 69.4%), but this figure describes the composition of the AR subgroup rather than prevalence (as shown in Table 1).

**TABLE 1:** Characteristics of AR participants (*n* = 683).

Characteristic	Category	Male (*n* = 157)	Female (*n* = 526)	Total (*n* = 683)	*χ* ^2^	*df*	*p*
Age (years)	Mean ± SD	38.25 ± 15.17	34.78 ± 13.45	35.57 ± 13.93	—	—	**0.018**
	Median (range)	37.00 (18–76)	33.00 (18–70)	34.00 (10–76)			
Residence	Urban	103 (65.6%)	371 (70.5%)	474 (69.4%)	1.382	1	0.240
	Rural	54 (34.4%)	155 (29.5%)	209 (30.6%)			
Smoking	Smoker	65 (41.4%)	80 (15.2%)	145 (21.2%)	49.602	1	**< 0.001**
	Non‐smoker	92 (58.6%)	446 (84.8%)	538 (78.8%)			
Family history of AR	Yes	74 (47.1%)	308 (58.6%)	382 (55.9%)	6.399	1	**0.011**
	No	83 (52.9%)	218 (41.4%)	301 (44.1%)			
Income	< 500 JD	73 (46.5%)	268 (51.0%)	341 (49.9%)	0.959	1	0.327
	≥ 500 JD	84 (53.5%)	258 (49.0%)	342 (50.1%)			
Occupation	Working	100 (63.7%)	277 (52.7%)	377 (55.2%)	5.951	1	**0.015**
	Not working	57 (36.3%)	249 (47.3%)	306 (44.8%)			
Education	University/Master	129 (82.2%)	441 (83.8%)	570 (83.5%)	0.246	1	0.620
	Less than University	28 (17.8%)	85 (16.2%)	113 (16.5%)			
Marital status	Married	99 (63.1%)	296 (56.3%)	395 (57.8%)	2.282	1	0.131
	Single	58 (36.9%)	230 (43.7%)	288 (42.2%)			
Asthma	Yes	31 (19.7%)	49 (9.3%)	80 (11.7%)	12.719	1	**< 0.001**
	No	126 (80.3%)	477 (90.7%)	603 (88.3%)			
Season of most exacerbation*	Spring	78 (49.7%)	302 (57.4%)	380 (55.6%)	10.737	4	**0.030**
	Summer	48 (30.6%)	117 (22.2%)	165 (24.2%)			
	Autumn	51 (32.5%)	142 (27.0%)	193 (28.3%)			
	Winter	44 (28.0%)	94 (17.9%)	138 (20.2%)			
	More than one season	44 (28.0%)	102 (19.4%)	146 (21.4%)			

*Note:* Values are *n* (%) unless otherwise indicated. *p*‐values are from Pearson *χ* ^2^ tests for categorical variables and the Mann–Whitney *U* test for age; bold *p*‐values indicate statistical significance at *p* < 0.05. Among all 1572 respondents, AR prevalence was 157/419 (37.5%) in men and 526/1153 (45.6%) in women (*χ* ^2^ = 8.31, *df* = 1, *p* = 0.004; OR = 1.40, 95% CI 1.11–1.76). The 69.4% urban figure describes the composition of the AR subgroup (474/683). Residence‐specific AR prevalence using all respondents is reported in the Results: 47.1% in urban residents (474/1006) vs. 36.9% in rural residents (209/566). Male AR patients were significantly more likely to report asthma than female AR patients (19.7% vs. 9.3%; *χ* ^2^ = 12.72, *df* = 1, *p* < 0.001; OR = 2.40, 95% CI 1.47–3.91 for male vs. female).

* Percentages are based on *n* = 683. Because participants could report symptoms in more than one season, percentages exceed 100%.

Several baseline characteristics differed significantly by sex. Male AR patients were older than female AR patients (mean 38.25 ± 15.17 vs. 34.78 ± 13.45 years; median 37 vs. 33; Mann–Whitney *U* = 36,163.5, Z = −2.36, *p* = 0.018). Male AR patients were also significantly more likely to report asthma (19.7% vs. 9.3%; *χ* ^2^ = 12.72, *df* = 1, *p* < 0.001; OR = 2.40, 95% CI 1.47–3.91) and to be current smokers (41.4% vs. 15.2%; *χ* ^2^ = 49.60, *df* = 1, *p* < 0.001; OR = 3.94, 95% CI 2.65–5.85). Female AR patients were significantly more likely to have a family history of AR (58.6% vs. 47.1%; *χ* ^2^ = 6.40, *df* = 1, *p* = 0.011; OR = 1.59, 95% CI 1.11–2.27). Male AR patients were also more likely to be working (63.7% vs. 52.7%; *χ* ^2^ = 5.95, *df* = 1, *p* = 0.015). Season of exacerbation also differed significantly by sex (*χ* ^2^ = 10.74, *df* = 4, *p* = 0.030). Residence, income, education, and marital status did not differ significantly between sexes (as shown in Table 1).

Among participants with AR, sneezing was the predominant symptom (64.0%). Other symptoms include rhinorrhoea (53.9%), nasal congestion and itching (52.9%), eye itching and tearing (40.0%), cough (21.8%), shortness of breath (40.8%), snoring (27.8%), headache (57.1%), and fatigue (48.2%) (as shown in Table 2). These symptoms mostly occurred during spring (55.6%) (as shown in Table 1). Dust (87.7%), pollen (49.2%), and smoking (48.3%) were the most common triggers (as shown in Table 3).

**TABLE 2:** AR symptoms by sex (AR subgroup, *n* = 683).

Symptom	Male (*n* = 157) *n* (%)	Female (*n* = 526) *n* (%)	Total (*n* = 683) *n* (%)	*χ* ^2^	*df*	*p*	OR (95% CI)
Sneezing	102 (65.0%)	335 (63.7%)	437 (64.0%)	0.086	1	0.769	0.95 (0.65–1.37)
Rhinorrhoea	93 (59.2%)	275 (52.3%)	368 (53.9%)	2.353	1	0.125	0.75 (0.53–1.08)
Nasal itching & congestion	69 (43.9%)	292 (55.5%)	361 (52.9%)	6.489	1	**0.011**	1.59 (1.11–2.28)
Ocular symptoms	58 (36.9%)	215 (40.9%)	273 (40.0%)	0.779	1	0.377	1.18 (0.82–1.71)
Cough	30 (19.1%)	119 (22.6%)	149 (21.8%)	0.876	1	0.349	1.24 (0.79–1.94)
Shortness of breath	63 (40.1%)	216 (41.1%)	279 (40.8%)	0.044	1	0.834	1.04 (0.72–1.50)
Snoring	63 (40.1%)	127 (24.1%)	190 (27.8%)	15.382	1	**< 0.001**	0.48 (0.33–0.69)
Headache	69 (43.9%)	321 (61.0%)	390 (57.1%)	14.395	1	**< 0.001**	2.00 (1.39–2.86)
Fatigue	65 (41.4%)	264 (50.2%)	329 (48.2%)	3.741	1	0.053	1.43 (0.99–2.05)

*Note:* Values are *n* (%) unless otherwise indicated. Bold *p*‐values indicate statistical significance at *p* < 0.05. The reference category for sex is male; odds ratios are for female vs. male. After Benjamini–Hochberg FDR correction: nasal itching/congestion, snoring, and headache remain significant; fatigue does not (*q* = 0.119).

Abbreviations: *χ* ^2^, Pearson chi‐square; CI, confidence interval; OR, odds ratio for female vs. male.

**TABLE 3:** Triggers of AR by sex (AR subgroup, *n* = 683).

Trigger*	Male (*n* = 157) *n* (%)	Female (*n* = 526) *n* (%)	Total (*n* = 683) *n* (%)	*χ* ^2^	*df*	*p*	OR (95% CI)
Dust	136 (86.6%)	463 (88.0%)	599 (87.7%)	0.219	1	0.640	1.14 (0.67–1.93)
Pollens	77 (49.0%)	259 (49.2%)	336 (49.2%)	0.002	1	0.966	1.01 (0.71–1.44)
Smoking (tobacco smoke)	61 (38.9%)	269 (51.1%)	330 (48.3%)	7.310	1	**0.007**	1.65 (1.15–2.37)
Fumes/perfume	61 (38.9%)	240 (45.6%)	301 (44.1%)	2.251	1	0.134	1.32 (0.92–1.90)
Molds	62 (39.5%)	237 (45.1%)	299 (43.8%)	1.522	1	0.217	1.26 (0.87–1.81)
Animal dander	32 (20.4%)	119 (22.6%)	151 (22.1%)	0.353	1	0.553	1.14 (0.74–1.77)
Food	14 (8.9%)	30 (5.7%)	44 (6.4%)	2.072	1	0.150	0.62 (0.32–1.20)
Medication	8 (5.1%)	22 (4.2%)	30 (4.4%)	0.240	1	0.624	0.81 (0.36–1.86)

*Note:* Values are *n* (%) unless otherwise indicated. Bold *p*‐values indicate statistical significance at *p* < 0.05. The reference category for sex is male; odds ratios are for female vs. male. After Benjamini–Hochberg FDR correction for eight trigger comparisons, no trigger survived FDR correction at *q* < 0.05; the smallest *q*‐value was for smoking (*q* = 0.056). All trigger findings should therefore be considered exploratory.

Abbreviations: *χ* ^2^, Pearson chi‐square; CI, confidence interval; OR, odds ratio for female vs. male.

* Triggers were self‐attributed and not confirmed by skin‐prick or specific IgE testing.

Analysis of AR symptoms revealed several significant sex‐based variations. Females reported a significantly higher prevalence of nasal itching and congestion compared to males (55.5% vs. 43.9%; *χ* ^2^ = 6.49, *df* = 1, *p* = 0.011; OR = 1.59, 95% CI 1.11–2.28). Additionally, headaches (61.0% vs. 43.9%; *χ* ^2^ = 14.40, *df* = 1, *p* < 0.001; OR = 2.00, 95% CI 1.39–2.86) were more common in female patients. However, male patients were more commonly affected by snoring than female patients (40.1% vs. 24.1%; *χ* ^2^ = 15.38, *df* = 1, *p* < 0.001; OR = 0.48, 95% CI 0.33–0.69). Fatigue was more common in females but did not reach statistical significance (50.2% vs. 41.4%; *χ* ^2^ = 3.74, *df* = 1, *p* = 0.053; OR = 1.43, 95% CI 0.99–2.05) (as shown in Table 2). After Benjamini–Hochberg FDR correction for nine symptom comparisons, nasal itching/congestion (*q* = 0.033), snoring (*q* < 0.001), and headache (*q* < 0.001) remained statistically significant. Fatigue did not survive FDR correction (*q* = 0.119) and should be considered exploratory (as shown in Table 2).

However, when comparing AR symptoms across general categories, sneezing, rhinorrhea, ocular symptoms, cough, and shortness of breath showed similar incidence rates in both sexes.

Self‐reported AR triggers were similar across sexes and included dust, pollen, mold, animal dander, and medications. However, smoking (tobacco smoke) was more triggering in female than male patients (51.1% vs. 38.9%; *χ* ^2^ = 7.31, *df* = 1, *p* = 0.007; OR = 1.65, 95% CI 1.15–2.37). On the other hand, male patients had a higher rate of food‐triggering AR compared to female patients (8.9% vs. 5.7%), although this difference was not statistically significant (*χ* ^2^ = 2.07, *df* = 1, *p* = 0.150; OR = 0.62, 95% CI 0.32–1.20) (as shown in Table 3). After Benjamini–Hochberg FDR correction for eight trigger comparisons, no trigger survived FDR correction at *q* < 0.05; the smallest *q*‐value was for smoking (*q* = 0.056). All trigger findings should therefore be considered exploratory (as shown in Table 3).

Table 4 and Figure 1 show the relationship between quality of life and the study variables. AR profoundly affected QOL across both sexes, particularly sleep (80.7%), concentration (66.2%), and work performance (57.2%). The sense of smell was also affected comparably in both sexes, with a 63.1% impact in males and a 62.5% impact in females.

**TABLE 4:** Impact of AR on quality of life by sex (AR subgroup, *n* = 683).

QOL item	Male (*n* = 157) *n* (%)	Female (*n* = 526) *n* (%)	Total (*n* = 683) *n* (%)	*χ* ^2^	*df*	*p*	OR (95% CI)
Daily activities affected	79 (50.3%)	265 (50.4%)	344 (50.4%)	0.000	1	0.989	1.00 (0.70–1.43)
Activities outside home affected	72 (45.9%)	290 (55.1%)	362 (53.0%)	4.174	1	**0.041**	1.45 (1.01–2.08)
Sleep quality affected	125 (79.6%)	426 (81.0%)	551 (80.7%)	0.146	1	0.703	1.09 (0.70–1.70)
Sense of smell affected	99 (63.1%)	329 (62.5%)	428 (62.7%)	0.013	1	0.908	0.98 (0.68–1.42)
Sense of taste affected	49 (31.2%)	175 (33.3%)	224 (32.8%)	0.233	1	0.629	1.10 (0.75–1.61)
Work/study performance affected	85 (54.1%)	306 (58.2%)	391 (57.2%)	0.804	1	0.370	1.18 (0.82–1.69)
Sick leave taken	51 (32.5%)	168 (31.9%)	219 (32.1%)	0.016	1	0.898	0.98 (0.67–1.43)
Concentration affected	101 (64.3%)	351 (66.7%)	452 (66.2%)	0.311	1	0.577	1.11 (0.77–1.62)
Appetite/weight affected	61 (38.9%)	171 (32.5%)	232 (34.0%)	2.170	1	0.141	0.76 (0.52–1.10)
Social relationships affected	58 (36.9%)	167 (31.7%)	225 (32.9%)	1.476	1	0.224	0.79 (0.55–1.15)
Prefer to be alone/isolated	75 (47.8%)	312 (59.3%)	387 (56.7%)	6.563	1	**0.010**	1.59 (1.11–2.28)
Embarrassment/shame	57 (36.3%)	180 (34.2%)	237 (34.7%)	0.232	1	0.630	0.91 (0.63–1.32)
Depression/frustration	52 (33.1%)	212 (40.3%)	264 (38.7%)	2.631	1	0.105	1.36 (0.94–1.98)
Increased stress/anxiety	67 (42.7%)	281 (53.4%)	348 (51.0%)	5.588	1	**0.018**	1.54 (1.08–2.21)
Irritability/emotional distress	87 (55.4%)	344 (65.4%)	431 (63.1%)	5.178	1	**0.023**	1.52 (1.06–2.19)

*Note:* Values are *n* (%) unless otherwise indicated. Bold *p*‐values indicate statistical significance at *p* < 0.05. The reference category for sex is male; odds ratios are for female vs. male. After Benjamini–Hochberg FDR correction: none of the QOL items survive FDR at *q* < 0.05; findings should be considered exploratory.

Abbreviations: *χ* ^2^, Pearson chi‐square; CI, confidence interval; OR, odds ratio for female vs. male.

**FIGURE 1:**
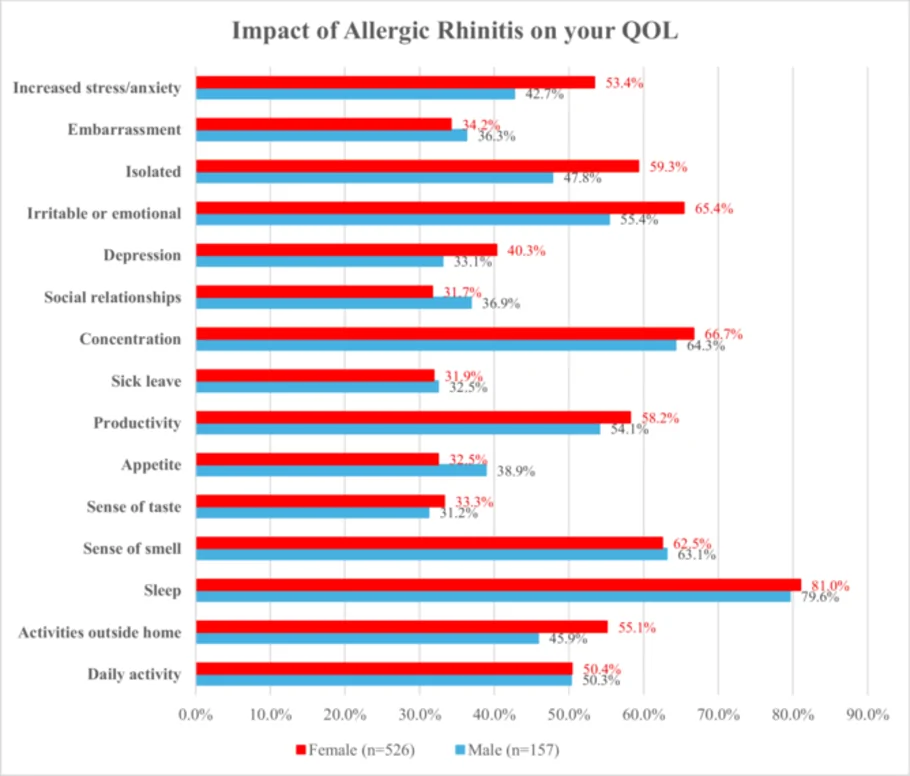
Impact of AR on your life during illness or symptoms for males and females.

However, critical sex differences emerged in psychological and social domains. Female participants were significantly more likely than males to experience increased irritability and emotional distress (65.4% vs. 55.4%; *χ* ^2^ = 5.18, *df* = 1, *p* = 0.023; OR = 1.52, 95% CI 1.06–2.19), to prefer social isolation during symptom episodes (59.3% vs. 47.8%; *χ* ^2^ = 6.56, *df* = 1, *p* = 0.010; OR = 1.59, 95% CI 1.11–2.28), to report increased stress/anxiety (53.4% vs. 42.7%; *χ* ^2^ = 5.59, *df* = 1, *p* = 0.018; OR = 1.54, 95% CI 1.08–2.21), and to report limitations in activities outside the home (55.1% vs. 45.9%; *χ* ^2^ = 4.17, *df* = 1, *p* = 0.041; OR = 1.45, 95% CI 1.01–2.08).

On the other hand, experiencing depression/frustration during the period of illness was reported by 33.1% of males and 40.3% of females, with no statistically significant difference between sexes (*χ* ^2^ = 2.63, *df* = 1, *p* = 0.105; OR = 1.36, 95% CI 0.94–1.98). Similarly, feelings of embarrassment were reported by 36.3% of males and 34.2% of females (*χ* ^2^ = 0.23, *df* = 1, *p* = 0.630; OR = 0.91, 95% CI 0.63–1.32), and social relationships were affected in 36.9% of males and 31.7% of females (*χ* ^2^ = 1.48, *df* = 1, *p* = 0.224; OR = 0.79, 95% CI 0.55–1.15), with no significant sex differences. After Benjamini–Hochberg FDR correction for 15 QOL comparisons, none of the QOL items remained statistically significant at *q* < 0.05. The smallest *q*‐value was for preference for social isolation (*q* = 0.150), followed by increased stress/anxiety (*q* = 0.135) and irritability/emotional distress (*q* = 0.115). All QOL findings should therefore be considered exploratory and hypothesis‐generating.

### 3.1 Adjusted Analyses

After adjustment for age, income, occupation, smoking, family history, residence, and education, female sex remained significantly associated with irritability/emotional distress (adjusted OR = 1.50, 95% CI 1.02–2.22, *p* = 0.039) and preference for social isolation (adjusted OR = 1.53, 95% CI 1.05–2.23, *p* = 0.028). Older age was inversely associated with irritability (adjusted OR = 0.98 per year, 95% CI 0.96–0.99, *p* < 0.001), and being employed was associated with higher odds of irritability (adjusted OR = 1.61, 95% CI 1.12–2.32, *p* = 0.010). Urban residence was associated with lower odds of social isolation (adjusted OR = 0.65, 95% CI 0.46–0.92, *p* = 0.015) (as shown in Tables 5 and 6).

**TABLE 5:** Multivariable logistic regression for irritability/emotional distress (AR subgroup, *n* = 683).

Variable	*B*	S.E.	Wald	*df*	*p*	Adjusted OR	95% CI
Female sex	0.408	0.198	4.253	1	**0.039**	1.503	1.020–2.215
Age	−0.025	0.007	12.600	1	**< 0.001**	0.975	0.962–0.989
Income (≥ 500 JD)	−0.145	0.183	0.621	1	0.431	0.865	0.604–1.240
Occupation (working)	0.478	0.186	6.579	1	**0.010**	1.613	1.119–2.323
Smoking	0.029	0.205	0.020	1	0.886	1.030	0.689–1.539
Family history of AR	0.005	0.167	0.001	1	0.975	1.005	0.725–1.395
Urban residence	−0.170	0.184	0.852	1	0.356	0.843	0.588–1.211
University education	−0.055	0.223	0.061	1	0.806	0.947	0.612–1.465
Constant	1.087	0.359	9.169	1	0.002	2.966	—

Model summary: Omnibus *χ* ^2^(8) = 26.317, *p* = 0.001; Hosmer–Lemeshow *χ* ^2^(8) = 4.601, *p* = 0.799; Nagelkerke *R* ^2^ = 0.052. Events per variable = 31.5 (smaller outcome category = 252 participants without irritability; eight predictor variables). Complete‐case analysis was used; there were no missing values for any covariate included. Reference categories: sex (male), income (≥ 500 JD), occupation (working), smoking (non‐smoker), family history of AR (no), residence (rural), and education (university or higher). The reference category for sex is male; odds ratios are for female vs. male. Maximum variance inflation factor = 1.48, indicating no collinearity concern.

**TABLE 6:** Multivariable logistic regression for social isolation (AR subgroup, *n* = 683).

Variable	*B*	S.E.	Wald	*df*	*p*	Adjusted OR	95% CI
Female sex	0.423	0.193	4.799	1	**0.028**	1.527	1.046–2.229
Age	0.002	0.007	0.119	1	0.730	1.002	0.989–1.016
Income (≥ 500 JD)	−0.246	0.178	1.907	1	0.167	0.782	0.552–1.108
Occupation (working)	0.111	0.179	0.383	1	0.536	1.117	0.787–1.586
Smoking	−0.192	0.199	0.938	1	0.333	0.825	0.559–1.218
Family history of AR	0.257	0.162	2.517	1	0.113	1.293	0.941–1.775
Urban residence	−0.437	0.179	5.965	1	**0.015**	0.646	0.455–0.917
University education	0.191	0.214	0.798	1	0.372	1.210	0.796–1.839
Constant	−0.031	0.347	0.008	1	0.930	0.970	—

Model summary: Omnibus *χ* ^2^(8) = 18.901, *p* = 0.015; Hosmer–Lemeshow *χ* ^2^(8) = 8.627, *p* = 0.375; Nagelkerke *R* ^2^ = 0.037. Events per variable = 37.0 (smaller outcome category = 296 participants without social isolation; eight predictor variables). Complete‐case analysis was used; there were no missing values for any covariate included. Reference categories: sex (male), income (≥ 500 JD), occupation (working), smoking (non‐smoker), family history of AR (no), residence (rural), and education (university or higher). The reference category for sex is male; odds ratios are for female vs. male. Maximum variance inflation factor = 1.48, indicating no collinearity concern. The reference category for sex is male; odds ratios are for female vs. male. Variables with significant *p*‐values (< 0.05) are bolded.

Sensitivity analysis: Adding self‐reported asthma as a covariate did not materially change the adjusted associations of female sex with irritability/emotional distress (adjusted OR = 1.53, 95% CI 1.04–2.27, *p* = 0.033) or with preference for social isolation (adjusted OR = 1.61, 95% CI 1.10–2.36, *p* = 0.015). Excluding the 80 participants with self‐reported asthma attenuated the association with irritability/emotional distress, which was no longer statistically significant (adjusted OR = 1.44, 95% CI 0.94–2.21, *p* = 0.091), but strengthened the association with preference for social isolation (adjusted OR = 1.84, 95% CI 1.21–2.79, *p* = 0.005). Asthma itself was not significantly associated with irritability (adjusted OR = 1.20, 95% CI 0.73–1.99, *p* = 0.474) or with social isolation (adjusted OR = 1.63, 95% CI 0.99–2.69, *p* = 0.053) in the adjusted models.

## 4 Discussion

This study aimed to evaluate the impact of allergic rhinitis on the quality of life of Jordanian adults. It is evident that allergic rhinitis significantly disrupts patients' daily routines, mental well‐being, and physical capabilities, leading to heightened personal and socioeconomic burdens. The growing recognition of the importance of quality of life in medical practice and research underscores the need to better understand how allergic rhinitis symptoms affect patients. This understanding can help physicians and researchers achieve more comprehensive outcomes, facilitate informed drug administration strategies, and ultimately improve the overall quality of life for individuals with Allergic Rhinitis.

In this study, women reported AR more frequently (526/1153, 45.6%) than men (157/419, 37.5%; *χ* ^2^ = 8.31, *df* = 1, *p* = 0.004), and the impact on quality of life was greater in women. We also found that females had significantly more irritability, poorer emotional well‐being, and a preference for aloneness or isolation than males. This finding concurred with those of Shariat M. et al., who found that AR significantly affects the QOL of females more than that of male patients [12]. Previous evidence also suggests that sex differences in allergic rhinitis may vary across the life course, with a shift from greater male prevalence in childhood toward greater female prevalence during adolescence and adulthood [8]. In addition, adult AR‐specific research has reported greater impairment in several quality‐of‐life domains among women, supporting the possibility that sex may influence not only the prevalence of AR but also its perceived functional and psychosocial burden [13]. Thus, the greater AR prevalence and some of the psychosocial differences observed among women in the present study are broadly consistent with previous literature rather than being entirely unique to the Jordanian population. Nevertheless, the specific pattern and magnitude of the observed associations may reflect characteristics of the study population and its sociocultural context.

Several mechanisms may potentially contribute to the observed differences in reported symptom burden and psychosocial effects between male and female participants. Biological factors, including sex‐related hormonal and immune differences, may influence inflammatory responses and symptom perception. Behavioral and healthcare‐utilization differences may also influence symptom recognition and reporting. Sociocultural factors may additionally shape how symptoms affect daily responsibilities, social interactions, and emotional responses. However, these mechanisms were not directly measured in the present study and therefore cannot be established as explanations for the observed associations. The persistence or attenuation of the sex differences after adjustment for available demographic and behavioral covariates should therefore be considered when interpreting these findings.

Several baseline differences between sexes are worth noting when interpreting the psychosocial findings. Male AR patients were significantly older (median 37 vs. 33 years, *p* = 0.018) and reported significantly higher rates of asthma (19.7% vs. 9.3%) and smoking (41.4% vs. 15.2%), whereas female AR patients were more likely to report a family history of AR (58.6% vs. 47.1%). Because age, smoking, asthma, and occupation are potential confounders of the association between sex and quality of life, all multivariable models were adjusted for these variables. The persistence of the female‐sex associations with irritability and social isolation after adjustment for these covariates suggests that the observed differences are not solely attributable to baseline demographic or clinical differences between sexes.

In our sample, AR prevalence was higher in urban than in rural residents (47.1% vs. 36.9%; *χ* ^2^ = 15.31, *df* = 1, *p* < 0.001; OR = 1.52, 95% CI 1.23–1.88), consistent with the findings of Shpakou A. et al. and Appiah‐Thompson P. et al. [14, 15]. However, because the survey used convenience sampling and urban residents were over‐represented, this finding should be interpreted cautiously and confirmed in population‐based surveys.

The most common aeroallergens identified in our study were dust and pollens. According to a 2015 study by Siwaporn Sapsaprang in Thailand, the most commonly reported triggers among Thai patients were dust mites and American cockroaches [16]. Another study by Peter Appiah‐Thompson in Ghana in 2023 found that dust mites were the most reported triggers [15]. On the other hand, Maher Maoua and his colleagues found that cotton dust was the most reported trigger [9].

Based on symptom frequency, our findings indicate that the most prevalent symptoms among patients were sneezing and rhinorrhoea. In 2012, another study by Mansoureh Shariat in Iran on quality of life in allergic rhinitis found that nasal congestion and rhinorrhoea were the most prevalent symptoms [12]. Also, a South African study by Devesh Ramdhani in 2021 found that nasal congestion was the most commonly reported symptom among AR patients in clinics [17]. Moreover, a study by Maher Maoua in Tunisia found that sneezing, itching, and nasal obstruction were the most commonly reported AR symptoms [9].

Shortness of breath was reported by 40.8% of participants with physician‐diagnosed AR, while asthma was reported by 80/683 (11.7%) of AR patients. The coexistence of AR and asthma is well documented, as they share a common inflammatory substrate and frequently coexist. A united‐airway approach may help interpret dyspnea in patients with AR [18]. However, the proportion of patients reporting shortness of breath (40.8%) was higher than the reported prevalence of asthma (11.7%), suggesting that other factors, such as nasal obstruction, postnasal drip, or undiagnosed asthma, may contribute to dyspnea in this population. Therefore, asthma comorbidity and other respiratory conditions should be considered when evaluating respiratory symptoms in patients with AR. Future studies should therefore incorporate systematic assessment of asthma and, where feasible, objective respiratory evaluation to clarify the contribution of lower‐airway comorbidity to the reported quality‐of‐life burden. In our study, male AR patients were significantly more likely to report asthma than female AR patients (19.7% vs. 9.3%, *p* < 0.001). Sensitivity analyses showed that the association between female sex and social isolation remained significant whether asthma was included as a covariate or participants with asthma were excluded, suggesting this finding is not driven by lower‐airway comorbidity. In contrast, the association between female sex and irritability was attenuated and no longer significant after excluding participants with asthma, suggesting that part of this signal may reflect coexisting asthma rather than AR alone.

In our study, we found that individuals experiencing symptoms of AR tend to become more irritable and experience changes in their emotional well‐being. They also tend to withdraw or feel isolated, particularly female patients. Depression, embarrassment, and social‐relationship impairment were reported by approximately one‐third of participants in both sexes. Our results suggest that such tendencies can negatively impact patients' overall quality of life across various aspects. According to a 2021 study conducted by Devesh Ramdhani and Tamaryn Daniller, patients faced numerous challenges, with activity limitations and nasal symptoms being the most prominent. A blocked nose emerged as the most troublesome symptom. Apart from the physical aspects, patients struggled with learning difficulties, decreased work productivity, and exclusion from social activities. Practical concerns, such as the constant need for tissues, added to their overall burden. Treatment costs and reduced work productivity further affected these individuals' socioeconomic status [17]. Accordingly, we found that more than half of males and females (54.1% and 58.2%, respectively) reported that AR affected their performance at work or in their studies. This finding was consistent with those of Maoua M et al., Small M et al., Vieira RJ et al., and Appiah‐Thompson P, who also found that AR significantly reduced productivity and QOL [9, 15, 19, 20]. Additionally, de la Hoz Caballer B et al. found that AR can cause a 27.8% decrease in productivity and daily activity, which is even more significant than chronic diseases such as hypertension and diabetes mellitus [21]. Moreover, Almalki ZA et al. reported that AR was associated with poorer academic performance in both sexes, with a greater impact reported among male students in their cohort [22].

In this study, we found that individuals with allergic rhinitis experienced limitations in their quality of life, particularly in aspects such as irritability, emotional well‐being, and social isolation. They also reported anxiety during symptoms, which affected their daily activities.

Interestingly, despite the significant differences in psychological domains, other aspects such as the prevalence of depression, feelings of embarrassment, concentration, and appetite changes were similarly high in both sexes. This suggests that while the expression of the AR burden may be sex‐specific, the core emotional toll of the disease is universally high, underscoring AR as a significant contributor to mental health strain across the population.

Additionally, the study suggests that individuals become more stressed and anxious during the period of illness or symptom onset. In another study in the US in 2018, individuals who have a history of seasonal allergies are more likely to experience mood disorders, anxiety disorders, and eating disorders. However, the study did not find any significant association between seasonal allergies and alcohol or substance use disorders after adjusting for socio‐demographic variables and tobacco use [23]. Moreover, a Taiwanese study conducted by Nian‐Sheng Tzeng in 2018 found that AR was significantly associated with an increased risk of having psychiatric diseases [24]. Additionally, AR affects appetite in 38.9% of males and 32.5% of females, with no significant association between sex and the effect of AR on appetite and eating behavior. On the other hand, Oh H et al. found that male patients had significantly more eating disorders compared with female patients [23].

In our survey, we found no significant relationship between sex and sleep quality. Nevertheless, 80.7% of participants reported that AR symptoms affected their sleep, indicating a substantial overall sleep burden despite no statistically significant sex difference. In 2008, Craig et al. conducted a study in the United States on sleep disorders in patients with AR, rhinosinusitis, and nasal polyposis. The study indicated that nasal congestion was among the most prevalent symptoms of these diseases, leading to breathing disorders during sleep. These sleep disorders were found to significantly affect patients' quality of life [25]. A study conducted in Brazil by Ines Cristina Camelo Nunes in 2009 revealed that patients with allergic rhinitis often experience a reduced quality of life due to the classic symptoms of the disease. The most prominent symptom, nasal obstruction, is associated with sleep disorders that can have a profound effect on mental health and learning [26]. Shariat M et al. also found a significant association between nasal congestion and sleep disorders [12]. In a 2012 study by Vanesa González‐Núñez, Patients with allergic rhinitis (AR) experienced a significant reduction in quality of life (QOL) due to the negative impact on sleep. A study has shown that AR patients are more likely to suffer from insomnia, snoring, and sleep apnea syndrome compared to the control group. AR affects all aspects of sleep. AR severity significantly affects nocturnal sleep duration, daytime sleepiness frequency, and time to fall asleep [27].

Overall, AR imposes a multidimensional impact on physical, emotional, and social aspects of life, with some exploratory sex‐related differences observed in the present population. Although the greater prevalence of AR among women and the greater reporting of selected psychosocial outcomes among women are broadly compatible with previous evidence, these findings should not be interpreted as demonstrating a universal female predominance in AR‐related psychosocial burden. Differences in sampling methods, measurement instruments, population characteristics, cultural context, symptom perception, and healthcare‐seeking behavior may contribute to variation between studies. In particular, the present study used online convenience sampling and a purpose‐designed, non‐validated questionnaire, and its cross‐sectional design does not permit causal inference. Therefore, the observed sex‐related associations should be considered exploratory and hypothesis‐generating. Further studies using representative samples, validated AR‐specific quality‐of‐life instruments, and objective measures of allergic disease are needed to determine whether these patterns are reproducible in Jordan and other Middle Eastern populations.

### 4.1 Implications for Clinical Practice and Public Health

The findings suggest that AR may impose a greater psychological and social burden on women than on men. If confirmed in adequately powered, representative studies, these signals would support a sex‐sensitive approach in clinical practice, including proactive screening of female AR patients for irritability, emotional distress, and social withdrawal. At present, however, because none of the quality‐of‐life findings survived FDR correction, these recommendations should be regarded as hypothesis‐generating rather than as grounds for changing clinical practice.

Furthermore, the universal finding that AR severely disrupts sleep, concentration, and work performance underscores the need to treat AR not merely as a nasal disorder, but as a systemic condition with far‐reaching socioeconomic consequences. Public health awareness campaigns should educate both the public and employers about the actual burden of AR to reduce stigma and foster supportive environments. In addition, intranasal corticosteroids remain first‐line therapy for persistent and moderate‐to‐severe AR, and concerns about corticosteroids can reduce adherence and limit improvement in quality of life [28]. Addressing steroid‐related concerns should therefore be considered part of a comprehensive approach to improving QOL in patients with AR. Finally, identifying dust and pollen as the dominant triggers in Jordan provides a clear target for environmental and public health interventions, such as promoting air‐quality monitoring and personalized allergen‐avoidance strategies.

#### 4.1.1 Study's Contribution to Previous Knowledge

This study offers insight into the overall quality of life (QOL) of allergic rhinitis (AR) in Jordan, highlighting dust and pollen as the most commonly reported triggers. Additionally, the higher prevalence of AR in Jordanian women aligns with global trends and provides a localized context. It highlights significant isolation preferences and stress during symptom onset, which were previously underexplored in the literature. Moreover, about two‐thirds of AR patients are in urban areas.

#### 4.1.2 Strengths, Limitations, and Future Directions

This study is characterized by a large convenience sample, focuses on the Jordanian population, and addresses a regional research gap to help authorities support AR patients.

However, it has several limitations. First, the questionnaire was purpose‐designed from existing instruments, piloted for face validity only (*n* = 20), and not formally validated. Because we did not establish reliability, internal consistency, or construct validity, the instrument should be considered a purpose‐designed questionnaire rather than a formally validated instrument. Second, it is based on an online self‐report questionnaire, and recruitment was via social media, which may lead to reporting bias and a non‐representative sample. Third, it lacks clinical data, such as IgE levels. Fourth, asthma was self‐reported by 11.7% of AR patients and was not objectively confirmed; this may have led to under‐ or over‐reporting and limits interpretation of respiratory symptoms, particularly given the higher proportion reporting shortness of breath (40.8%). Male AR patients reported asthma more frequently than female AR patients (19.7% vs. 9.3%, *p* < 0.001). Sensitivity analyses showed that the association between female sex and social isolation was robust to the inclusion or exclusion of asthma, whereas the association with irritability was attenuated after excluding participants with asthma and should therefore be interpreted with caution. Fifth, the study did not address AR severity classification, which limits analysis of how severity affects QOL. Sixth, the exclusion of participants with psychological problems likely biased psychological findings toward the null and limits generalizability to patients with comorbid psychiatric conditions. Seventh, the male AR subgroup (*n* = 157) had limited power; findings in this subgroup should be interpreted cautiously. Eighth, the sample size does not attenuate systematic bias. Findings should therefore be considered exploratory and hypothesis‐generating. In addition, the two multivariable regression outcomes were selected following the initial unadjusted analyses because they showed notable sex differences. Although adjustment for potential confounders strengthened the assessment of these associations, the model selection was post hoc, and the resulting adjusted *p*‐values were not independently corrected for multiplicity. These findings should therefore be regarded as exploratory signals requiring confirmation in future studies with prespecified outcomes and appropriately powered analyses. Finally, because the survey was designed so that only participants with physician‐diagnosed AR completed the symptom, trigger, and quality‐of‐life sections, no non‐AR comparator data were available for these outcomes. This limits our ability to determine whether the observed QOL burden is specific to AR or reflects broader population‐level factors. Future studies should include a non‐AR comparison group to provide a baseline for QOL measures.

Building upon the findings and limitations of this study, several key avenues for future research are recommended. To establish causality and understand the long‐term course of quality‐of‐life impairment, longitudinal studies are essential. Furthermore, integrating objective clinical measures, such as IgE levels, allergy testing, and ARIA‐based severity classification, with subjective quality‐of‐life reports would provide a more holistic understanding of the disease burden. For example, device‐derived, examiner‐independent parameters have been shown to complement symptom‐based assessment in atopic dermatitis [29]. Future AR studies could integrate similar objective measures with subjective quality‐of‐life reports.

The sex‐related findings also warrant further investigation through representative and comparative studies. Qualitative studies could explore the lived experiences and sociocultural factors that may contribute to differences in psychological distress and social isolation, while biomedical research could investigate potential biological mechanisms underlying sex‐related differences in allergic inflammation. Comparative studies across Jordan and other Middle Eastern populations would also help determine whether the observed patterns are consistent across settings or are partly specific to the local cultural and environmental context.

## 5 Conclusion

The study analyzed 1572 individuals and found that 683 of them had allergic rhinitis (AR). Females were more likely to have AR than males, and most AR patients lived in urban areas. The study revealed that patients who preferred solitude or isolation during AR symptoms experienced increased levels of stress and anxiety. Additionally, patients became more irritable and emotional during the illness or at symptom onset. Moreover, AR negatively affected sleep, work performance, study performance, and daytime concentration. Depression, embarrassment, and social‐relationship impairment were reported by approximately one‐third of participants in both sexes. Females reported statistically significant unadjusted symptoms of irritability, emotional well‐being, and preference for aloneness or isolation compared to males; however, these findings did not survive FDR correction and should be considered exploratory. After adjustment for age, income, occupation, smoking, family history, residence, and education, female sex was associated with higher odds of irritability/emotional distress (adjusted OR = 1.50, 95% CI 1.02–2.22, *p* = 0.039) and preference for social isolation (adjusted OR = 1.53, 95% CI 1.05–2.23, *p* = 0.028); however, these associations should be considered exploratory because the outcomes were selected after the initial analyses and the adjusted *p*‐values were not independently corrected for multiplicity. These findings provide signals for further investigation rather than evidence for sex‐specific changes in clinical practice.

## Author Contributions

**Hasan Ibrahim Al‐Balas:** conceptualization, methodology, software, data curation, investigation, validation, formal analysis, supervision, visualization, project administration, resources, writing – review and editing. **Almu'atasim Khamees:** conceptualization, methodology, data curation, investigation, writing – review and editing, validation, visualization. **Shatha Khaled Al Saoud:** data curation, investigation, writing – original draft. **Sajeda Ahmad Aldakhiel:** investigation, data curation, writing – original draft. **Wasan Abdallah Bani Issma'eel:** data curation, investigation, writing – original draft. **Farah Nayef Alzu'bi:** data curation, investigation, writing – original draft. **Nour Ahmad Mistareehi:** data curation, investigation, writing – original draft. **Majd Mohammed Qashou:** data curation, investigation, writing – original draft. **Sireen Kamal Ayasrah:** data curation, investigation, writing – original draft.

## Funding

The authors have nothing to report.

## Ethics Statement

The research has been approved by the Institutional Review Board (IRB) of Yarmouk University, Irbid, Jordan, number (IRB/2023/277). Participation was voluntary, and all participants provided informed consent electronically before completing the questionnaire. No personally identifying information was collected. The study was conducted in accordance with the ethical principles of the Declaration of Helsinki.

## Conflicts of Interest

The authors declare no conflicts of interest.

## Policy on Using ChatGPT and Similar AI Tools

The authors did not use any AI software or AI‐assisted tools in the writing of this manuscript.

## Supporting information


Supporting File

## Data Availability

The data that support the findings of this study are available from the corresponding author upon reasonable request.
