# Supplementary material for: Sex‐Specific Differences in Symptomatology and Quality of Life Impact of Allergic Rhinitis: A Cross‐Sectional Study
Source: Immun Inflamm Dis. 2026 Oct 22;14(10):e70565. doi: 10.1002/iid3.70565 (PMC13689530; doi:10.1002/iid3.70565)
Supplement: Supplementary file 1 [file IID3-14-e70565-s001.docx]

STROBE Statement—checklist of items that should be included in reports of observational studies

|  | Item No. | Recommendation | Page  No. | Relevant text from manuscript |
| --- | --- | --- | --- | --- |
| **Title and abstract** | 1 | (*a*) Indicate the study’s design with a commonly used term in the title or the abstract | 4 | Line 96 |
|  |  | (*b*) Provide in the abstract an informative and balanced summary of what was done and what was found | 4 | Lines 99-121 |
| Introduction | | | |  |
| Background/rationale | 2 | Explain the scientific background and rationale for the investigation being reported | 5-6 | Lines 127-159 |
| Objectives | 3 | State specific objectives, including any prespecified hypotheses | 6 | Lines 161-163 |
| Methods | | | |  |
| Study design | 4 | Present key elements of study design early in the paper | 6 | Lines 165-172 |
| Setting | 5 | Describe the setting, locations, and relevant dates, including periods of recruitment, exposure, follow-up, and data collection | 6 | Lines 165-172 |
| Participants | 6 | (*a*) *Cohort study*—Give the eligibility criteria, and the sources and methods of selection of participants. Describe methods of follow-up  *Case-control study*—Give the eligibility criteria, and the sources and methods of case ascertainment and control selection. Give the rationale for the choice of cases and controls  *Cross-sectional study*—Give the eligibility criteria, and the sources and methods of selection of participants | 6 | Lines 174-178 |
|  |  | (*b*) *Cohort study*—For matched studies, give matching criteria and number of exposed and unexposed  *Case-control study*—For matched studies, give matching criteria and the number of controls per case | NA | This is a cross-sectional study. |
| Variables | 7 | Clearly define all outcomes, exposures, predictors, potential confounders, and effect modifiers. Give diagnostic criteria, if applicable | 6 | Lines 165-178 |
| Data sources/ measurement | 8* | For each variable of interest, give sources of data and details of methods of assessment (measurement). Describe comparability of assessment methods if there is more than one group | 6 | Lines 165-178 |
| Bias | 9 | Describe any efforts to address potential sources of bias | 6 | Lines 165-170 |
| Study size | 10 | Explain how the study size was arrived at | 6 | Lines 165-170 |

Continued on next page

| Quantitative variables | 11 | Explain how quantitative variables were handled in the analyses. If applicable, describe which groupings were chosen and why | 6 | Lines 180-183 |
| --- | --- | --- | --- | --- |
| Statistical methods | 12 | (*a*) Describe all statistical methods, including those used to control for confounding | 6 | Lines 180-183 |
|  |  | (*b*) Describe any methods used to examine subgroups and interactions | 6 | Lines 180-183 |
|  |  | (*c*) Explain how missing data were addressed | NA | No missing data |
|  |  | (*d*) *Cohort study*—If applicable, explain how loss to follow-up was addressed  *Case-control study*—If applicable, explain how matching of cases and controls was addressed  *Cross-sectional study*—If applicable, describe analytical methods taking account of sampling strategy | 6 | Lines 180-183 |
|  |  | (*e*) Describe any sensitivity analyses | 6 | Lines 180-183 |
| Results | | | | |
| Participants | 13* | (a) Report numbers of individuals at each stage of study—eg numbers potentially eligible, examined for eligibility, confirmed eligible, included in the study, completing follow-up, and analysed | 7 | Lines 188-195 |
|  |  | (b) Give reasons for non-participation at each stage | NA | This is a cross-sectional study. |
|  |  | (c) Consider use of a flow diagram | NA | This is a cross-sectional study. |
| Descriptive data | 14* | (a) Give characteristics of study participants (eg demographic, clinical, social) and information on exposures and potential confounders | 7 | Lines 188-195 |
|  |  | (b) Indicate number of participants with missing data for each variable of interest | NA | No missing data |
|  |  | (c) *Cohort study*—Summarise follow-up time (eg, average and total amount) | NA | This is a cross-sectional study. |
| Outcome data | 15* | *Cohort study*—Report numbers of outcome events or summary measures over time | NA | This is a cross-sectional study. |
|  |  | *Case-control study—*Report numbers in each exposure category, or summary measures of exposure | NA | This is a cross-sectional study. |
|  |  | *Cross-sectional study—*Report numbers of outcome events or summary measures | 7-8 | Lines 188-229 |
| Main results | 16 | (*a*) Give unadjusted estimates and, if applicable, confounder-adjusted estimates and their precision (eg, 95% confidence interval). Make clear which confounders were adjusted for and why they were included | 7-8 | Lines 188-229 |
|  |  | (*b*) Report category boundaries when continuous variables were categorized | 7-8 | Lines 188-229 |
|  |  | (*c*) If relevant, consider translating estimates of relative risk into absolute risk for a meaningful time period | NA | NA |

Continued on next page

| Other analyses | 17 | Report other analyses done—eg analyses of subgroups and interactions, and sensitivity analyses | 7-8 | Lines 188-229 |
| --- | --- | --- | --- | --- |
| Discussion | | | | |
| Key results | 18 | Summarise key results with reference to study objectives | 8 | Lines 234-240 |
| Limitations | 19 | Discuss limitations of the study, taking into account sources of potential bias or imprecision. Discuss both direction and magnitude of any potential bias | 12-13 | Lines 355-366 |
| Interpretation | 20 | Give a cautious overall interpretation of results considering objectives, limitations, multiplicity of analyses, results from similar studies, and other relevant evidence | 8-13 | Lines 234-366 |
| Generalisability | 21 | Discuss the generalisability (external validity) of the study results | 8-13 | Lines 234-366 |
| Other information | |  | | |
| Funding | 22 | Give the source of funding and the role of the funders for the present study and, if applicable, for the original study on which the present article is based | 13 | Lines 383-384 |

*Give information separately for cases and controls in case-control studies and, if applicable, for exposed and unexposed groups in cohort and cross-sectional studies.

**Note:** An Explanation and Elaboration article discusses each checklist item and gives methodological background and published examples of transparent reporting. The STROBE checklist is best used in conjunction with this article (freely available on the Web sites of PLoS Medicine at http://www.plosmedicine.org/, Annals of Internal Medicine at http://www.annals.org/, and Epidemiology at http://www.epidem.com/). Information on the STROBE Initiative is available at www.strobe-statement.org.
